# Effect of Manufacturing Technique and Cementation Protocol on the Load-Bearing Capacity of 3D-Printed Zirconia Molar Crowns

**DOI:** 10.3390/bioengineering13090963

**Published:** 2026-08-23

**Authors:** Felix Oßwald, Franz Sebastian Schwindling, Stefan Rues, Martin Rosentritt, Laura Haas, Angelika Rauch

**Affiliations:** 1 Department of Prosthodontics, Medical University Innsbruck, 6020 Innsbruck, Austria; 2 Department of Prosthodontics, Heidelberg University Hospital, 69120 Heidelberg, Germany; 3 Department of Prosthetic Dentistry, UKR University Hospital Regensburg, 93042 Regensburg, Germany

**Keywords:** additive manufacturing, 3D-printed zirconia, zirconia crowns, lithography-based ceramic manufacturing, fracture resistance, adhesive cementation, conventional cementation, dental ceramics

## Abstract

This study evaluated the influence of the manufacturing method and cementation protocol on the fracture load of zirconia molar crowns. A total of 32 full-contour crowns with a wall thickness of 1 mm were fabricated from 3 mol% yttria-stabilized tetragonal zirconia polycrystal (3Y-TZP). Sixteen crowns were produced by 3D printing (LithaCon 3Y 210, Lithoz) and 16 by milling (e.max ZirCAD LT, Ivoclar Vivadent). Within each manufacturing group, eight crowns were adhesively luted with a dual-cure resin cement (Bifix QM, VOCO) and eight were conventionally cemented with a glass ionomer cement (Ketac Cem, Solventum). All crown–die complexes underwent thermomechanical aging (6000 thermal cycles between 5 °C/55 °C; 1.2 × 10^6^ cycles at 50 N) as a correlate of five years of clinical service, followed by axial loading to failure. The statistical analysis was a two-way ANOVA on log10-transformed fracture loads with manufacturing and cement as fixed effects (α = 0.05). All crowns survived thermomechanical loading without failure. Milled zirconia crowns showed significantly higher fracture loads when adhesively luted (7162 ± 1294 N) compared with conventional cementation (4021 ± 1126 N; *p* < 0.001). In contrast, no significant differences were observed between adhesive (4986 ± 1301 N) and conventional cementation (5581 ± 1618 N) for 3D-printed zirconia crowns. Additively manufactured crowns exhibited greater variability in fracture loads (broader interquartile ranges) than their milled counterparts. Two-way ANOVA detected a significant Manufacturing × Cement interaction (*p* < 0.001), indicating that the effect of cementation differed by manufacturing route. Adhesive cementation increased fracture load for milled zirconia, whereas no statistically significant cementation effect was detected for 3D-printed zirconia under the present conditions. Given the sample size and the observed variability, the study was powered to detect very large effects but not medium or small ones; therefore, this non-significant difference should not be interpreted as proof of no effect.

## 1. Introduction

Digital workflows have gained importance in restorative dentistry, driven by the demand for more efficient treatment processes and improved patient comfort. Zirconia is widely used in this context due to its favorable mechanical properties, excellent biocompatibility, and suitability for minimally invasive preparation designs [1,2,3,4,5].

Traditionally, zirconia restorations have been fabricated using a subtractive approach, i.e., milling from pre-sintered blanks [6,7]. However, recent advancements in additive manufacturing have introduced 3D printing of zirconia as a promising alternative [8,9,10,11]. This technique offers several advantages, including the absence of tooling stress, greater geometric freedom, and minimized material waste [12,13,14,15,16]. Nonetheless, the mechanical performance of 3D-printed zirconia restorations remains insufficiently understood, and further investigation is required before widespread clinical adoption.

Load-bearing capacity is critical for the long-term success of posterior crowns, which must withstand masticatory forces of up to approximately 1000 N [17,18]. The cementation protocol is therefore an important clinical decision. Two strategies were compared: adhesive resin cementation and conventional glass-ionomer (GI). Adhesive luting can improve fracture resistance for weaker ceramic types such as glass-ceramics [19]. In combination with strong materials like milled zirconia, conventional cements perform well and remain a clinically relevant option for retentive preparations—particularly in posterior teeth under suboptimal moisture control—because of their low technique sensitivity [20]. Clinical reports show acceptable survival of zirconia crowns cemented with GI in such indications [21,22,23,24], and in vitro data support adequate mechanical performance with high-strength ceramics [25,26].

By contrast, several studies report that the flexural strength of 3D-printed zirconia is significantly lower than that of milled zirconia [27,28,29,30]. Therefore, it is a clinically relevant question whether the cement type affects the load-bearing capacity of 3D-printed zirconia molar crowns. The null hypothesis was that fracture resistance would not differ by the manufacturing process (milling or 3D printing) or the cementation regime (adhesive or conventional).

## 2. Materials and Methods

### 2.1. 3D-Printed Zirconia Crowns

**Figure 1:**
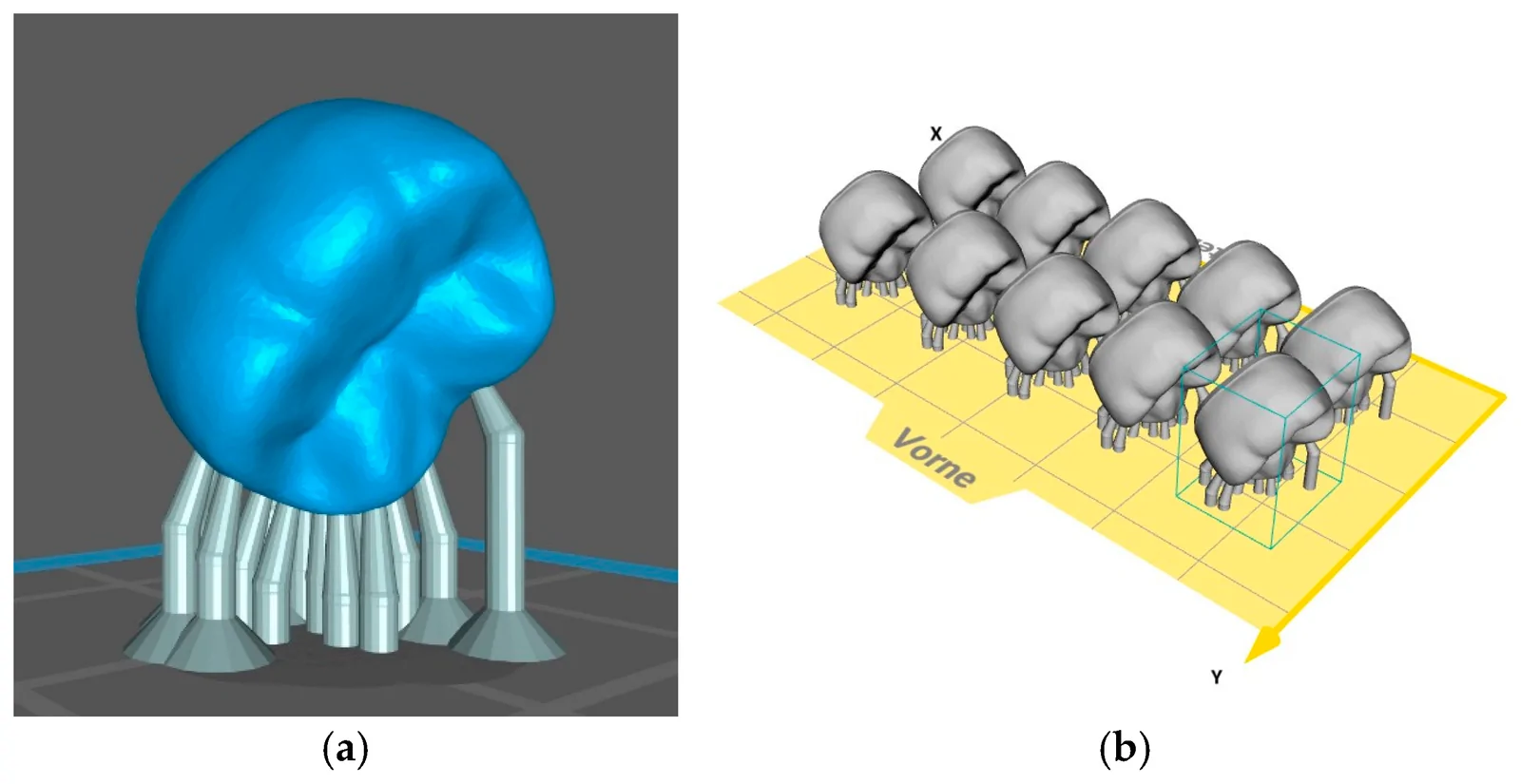
Representative 3D printing setup for a molar crown: (**a**) crown with column supports attached to the axial walls at the cervical third, predominantly on the mesio-lingual aspect, to avoid functional cusp tips and central fossae; (**b**) arrangement of the specimens on the build platform.

Full-contour crowns were designed using Dental Designer (version not recorded, 3Shape A/S, Copenhagen, Denmark), with a cement gap of 80 µm along the internal surface and a uniform wall thickness of 1 mm. Each crown was oriented as shown in Figure 1. Column supports were attached to the axial walls at the cervical third, avoiding functional cusp tips and central fossae. Supports were concentrated on the mesio-lingual aspect to keep them away from anticipated loading contacts.

Sixteen crowns (*n* = 16) were fabricated using lithography-based ceramic manufacturing (LCM), as previously described [16,31,32]. A printable 3Y-TZP zirconia–resin suspension (LithaCon 3Y 210, Lithoz GmbH, Vienna, Austria) was used with a CeraFab 7500 printer (Lithoz GmbH, Vienna, Austria). This material includes di- and monofunctional methacrylates, a reactive photoinitiator, a dispersant, an absorber, a non-reactive diluent, and approximately 48 vol% zirconia powder (D50 ~ 0.4 µm). The reported biaxial flexural strength exceeds 1200 MPa [27]. The 16 3D-printed crowns were produced in two independent print builds. Run parameters were as follows: layer height of 25 µm; lateral resolution of 40 µm; contour offset of −40 µm; scaling factors of 1.27 (XY) and 1.30 (Z); DLP energy of 70 mJ/cm^2^; light intensity of 55 mW/cm^2^; tilt-up velocity of 20°/s; rotation velocity of 120°/s (360°); and ambient temperature of 25 °C.

After printing, the green bodies (containing the resin matrix) were cleaned of slurry remnants using LithaSol 30 (Lithoz GmbH, Vienna, Austria) under air pressure. Binder removal was performed by heating to below 600 °C, followed by sintering to full density at 1450 °C in a single thermal cycle using a high-temperature furnace (LHT 02/16, Nabertherm GmbH, Lilienthal, Germany). Supports were left in place during sintering to allow vertical placement of the crowns. All 3D-printed crowns were sintered in one furnace cycle. Thereafter, supports were removed with a dental diamond bur (bur number K6974104220, Komet Dental, Gebr. Brasseler GmbH & Co. KG, Lemgo, Germany); support scars were not polished. No additional polishing of the external surface was performed. Intaglio surfaces were air-abraded per the cementation protocol (see Section 2.3).

### 2.2. Milled Zirconia Crowns

Control zirconia restorations were produced using conventional milling. Restorations (thickness: 1 mm) were designed using identical marginal and internal fit parameters as described for LCM zirconia. The restorations were milled from pre-sintered 3Y-TZP zirconia blanks (e.max ZirCAD LT A2, Lot No. Z02BNB; Ivoclar Vivadent AG, Schaan, Liechtenstein) using a 5-axis milling unit (PM7; Ivoclar Vivadent AG, Schaan, Liechtenstein) and sintered according to the manufacturer’s instructions.

### 2.3. Cementation/Adhesive Luting Protocol

The intaglio surfaces of all crowns were sandblasted with 50 µm alumina particles at 1.5 bar, steam-cleaned, and then subjected to ultrasonic cleaning in 99% isopropanol for 3 min. The crowns were dried using compressed air. The STL file of the “prepared” tooth was reproduced 32 times from 3D-printed resin (V-print model, VOCO). The die surfaces were sandblasted using 50 µm alumina at 1.0 bar pressure and steam-cleaned.

For the conventional cementation protocol, a glass-ionomer cement (GI, Ketac Cem, Solventum, Seefeld, Germany) was used, representing a clinically established luting option for zirconia restorations with retentive preparation designs. During setting, a constant seating pressure was applied for 5 min to ensure proper adaptation. For the adhesive groups, the intaglio zirconia surfaces were primed with Ceramic Bond (Ceramic Bond, VOCO GmbH, Cuxhaven, Germany), which was applied to the crown interiors for 60 s and air-dried with oil-free air. Ceramic Bond is a multi-purpose primer containing 3-methacryloxypropyltrimethoxysilane (3-MPTS). In response to a request for clarification, the manufacturer confirmed in writing that the product additionally contains 10-methacryloyloxydecyl dihydrogen phosphate (10-MDP; CAS No. 85590-00-7) at a concentration of <5 vol% [33]. Crowns were then luted with a dual-cure resin cement (Bifix QM, VOCO GmbH, Cuxhaven, Germany). Final polymerization was achieved by light-curing each surface for 20 s using a high-intensity LED curing unit.

### 2.4. Mechanical Testing

To simulate five years of clinical service, all specimens underwent thermomechanical loading consisting of 6000 thermal cycles between 5 °C and 55 °C (2 min/cycle, distilled water) and 1.2 × 10^6^ mechanical cycles at 50 N. Subsequently, the crowns were loaded to failure in a universal testing machine (Z010, Zwick, Ulm, Germany) to determine the fracture force. The force was applied to the center of the crowns using a steel ball with a diameter of 12 mm and a crosshead speed of 1 mm/min. To achieve a more uniform stress distribution, a 1 mm-thick tin foil was placed between the crown and the steel ball. Due to plastic deformation of the foil, the load was transferred simultaneously to the cuspal contact areas of the crown, resulting in five contact points corresponding to the number of cusps.

Failure was defined as either (i) a drop of ≥10% in the maximum recorded load or (ii) the occurrence of an acoustic signal detected via a microphone or headphones during testing. In addition, all force–displacement curves were reviewed after testing to verify the exact point of fracture.

### 2.5. Gross Post-Test Failure Classification

Based on the test records, the gross post-test condition of each crown was descriptively classified as either (i) crown fracture with fragment separation or (ii) crown crack without fragment separation. The extent of fragmentation and damage to the resin dies was not systematically assessed. This classification was not intended as a fractographic analysis and did not permit the determination of fracture origins or crack-propagation pathways.

### 2.6. Statistical Analysis

The primary analysis was a two-way ANOVA with manufacturing (milled vs. 3D-printed) and cement (adhesive vs. conventional) as fixed factors, including their interaction. Because the raw fracture load distributions were skewed and showed unequal variances, fracture loads were log10-transformed before analysis. Model assumptions were evaluated for the two-way ANOVA fitted to the log10-transformed fracture loads. Normality of the studentized residuals was assessed using the Shapiro–Wilk test and inspection of a Q–Q plot. Homogeneity of variance across the four Manufacturing × Cement cells was assessed using Levene’s test. A scale–location plot of the absolute studentized residuals against the fitted values was additionally inspected. Model results are reported as F values, degrees of freedom, *p* values, and partial η^2^. When the Manufacturing × Cement interaction was significant, simple effects were evaluated using estimated marginal means with Bonferroni adjustment. The significance level was set at α = 0.05 (SPSS Statistics Version 31.0, IBM, Armonk, NY, USA).

### 2.7. Sample Size and Sensitivity Analysis

The sample size was selected based on previous comparable investigations [34]. To characterize the sensitivity of the achieved four-group design, a sensitivity analysis based on the achieved sample size was performed. For a four-group design with α = 0.05, approximately 19 specimens per group (*N* ≈ 76) would be required to detect a large omnibus effect of Cohen’s f = 0.40 with 80% power. With the achieved sample size of *n* = 8 per group (*N* = 32), 80% power was attained only for very large omnibus effects of approximately Cohen’s f = 0.65. Thus, the study had limited sensitivity to medium and smaller effects.

## 3. Results

**Figure 2:**
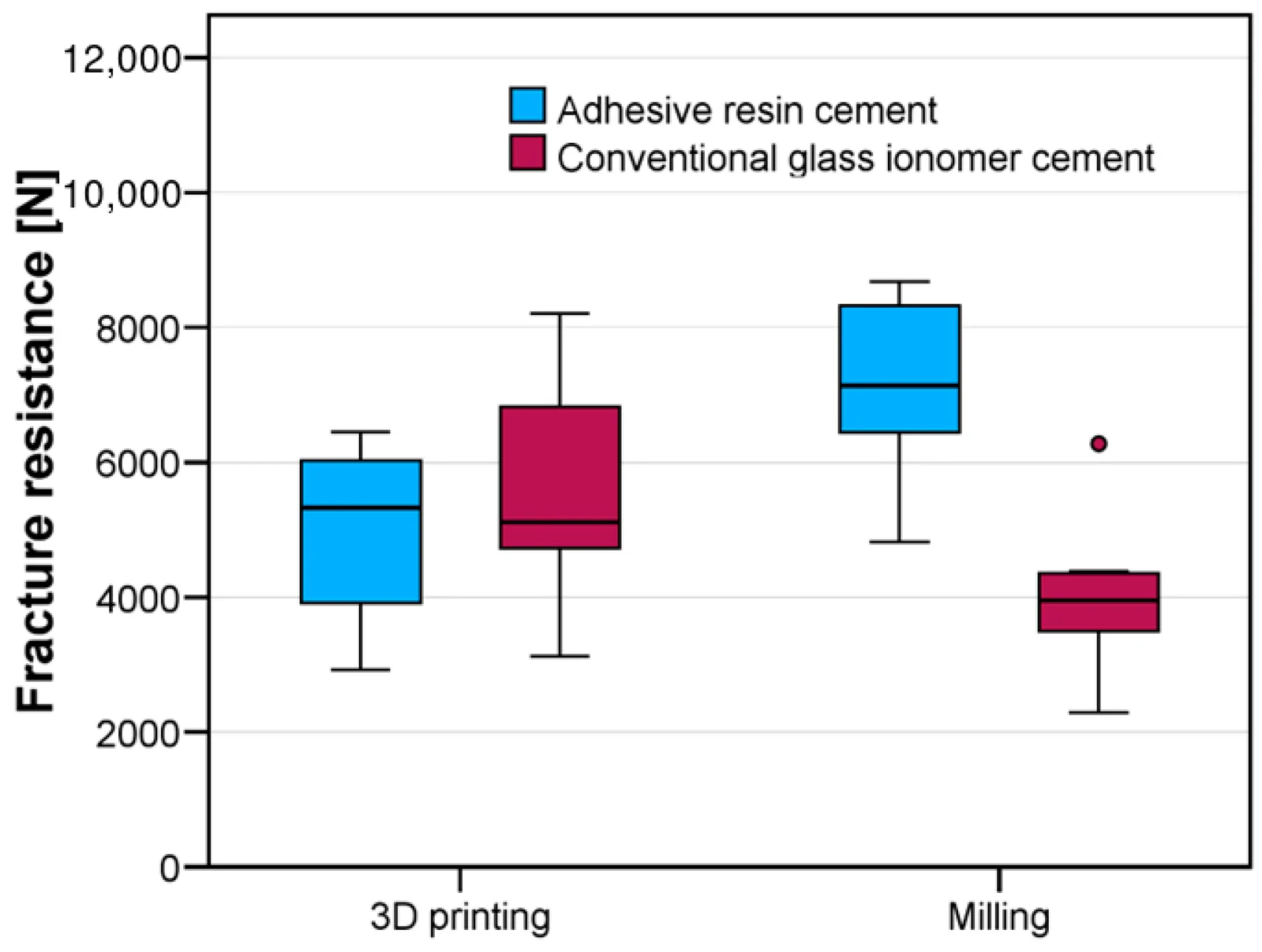
Boxplot—distribution of fracture resistance within the experimental groups.

All crowns survived artificial aging. The distribution of fracture loads is shown in Figure 2.

As shown in Table 1, adhesively luted milled zirconia crowns exhibited the highest mean fracture load among all groups (7162 ± 1294 N), with fracture forces ranging from 4814 to 8683 N. This group exhibited an interquartile range (IQR) of 2015 N. In contrast, milled crowns with conventional cementation required notably lower loads to fracture, with a mean fracture load of 4021 ± 1126 N (range: 2289–6275 N; IQR = 881 N).

In 3D-printed zirconia crowns, adhesive luting produced a mean fracture load of 4986 ± 1301 N (range: 2930–6448 N; IQR = 2195 N), whereas conventional cementation achieved a mean of 5581 ± 1618 N (range: 3126–8207 N; IQR = 2416 N). Gross post-test classification showed crown fractures with fragment separation in 31 of the 32 specimens. All crowns in both 3D-printed groups and in the milled-conventional group exhibited fracture with fragment separation. In the milled-adhesive group, seven crowns exhibited fracture with fragment separation, whereas one crown showed a crack without fragment separation.

### Factorial Analysis, Effect Sizes and Sensitivity Analysis

The two-way ANOVA on log10-transformed fracture loads revealed a significant Manufacturing × Cement interaction, F(1, 28) = 13.650, *p* < 0.001, partial η^2^ = 0.328. The main effect of cement was significant, F(1, 28) = 6.582, *p* = 0.016, partial η^2^ = 0.190, whereas the main effect of manufacturing was not, F(1, 28) = 0.088, *p* = 0.769, partial η^2^ = 0.003. Because of the significant interaction, interpretation focused on simple effects based on estimated marginal means with Bonferroni adjustment. Within milled crowns, adhesive cementation increased fracture load by a factor of 1.82 compared with conventional cementation (95% CI 1.38–2.39; *p* < 0.001). Within 3D-printed crowns, no statistically significant difference was detected between adhesive and conventional cementation (ratio 0.90, 95% CI 0.68–1.18; *p* = 0.431). The latter confidence interval indicates that effects ranging from a 32% decrease to an 18% increase remain compatible with the data; therefore, the result does not provide evidence of equivalence.

Assumption checks supported the use of factorial ANOVA on log10-transformed fracture loads. Studentized residuals showed no statistically significant departure from normality (Shapiro–Wilk W = 0.960, *p* = 0.277), and Levene’s test did not indicate heterogeneity of variance across the four Manufacturing × Cement cells, F(3, 28) = 0.546, *p* = 0.655. Visual inspection of the Q–Q and scale–location plots did not reveal marked systematic deviations.

The sensitivity analysis of the achieved four-group design indicated that 80% power was attained only for very large omnibus effects of approximately Cohen’s f = 0.65. The observed Manufacturing × Cement interaction corresponded to Cohen’s f ≈ 0.70, whereas the main effects of cement and manufacturing corresponded to f ≈ 0.48 and f ≈ 0.06, respectively. These effect-size estimates are reported descriptively and should not be compared directly with the omnibus sensitivity threshold. Overall, the limited sensitivity to medium and smaller effects means that a more modest cementation effect within the 3D-printed crowns cannot be excluded.

## 4. Discussion

This study found that the effect of cementation on load-bearing capacity depends on the zirconia manufacturing technique. Adhesive cementation significantly increased the load-bearing capacity of milled zirconia crowns, whereas no statistically significant benefit was detected for 3D-printed crowns. Therefore, the null hypothesis was partially rejected.

### 4.1. Cementation Effects and Clinical Interpretation

The higher fracture load obtained with adhesive cementation in milled zirconia is consistent with the combined effects of micromechanical retention after airborne-particle abrasion and the chemical interaction between 10-MDP and zirconia. Together, these mechanisms may reduce interfacial sliding and contribute to more effective load transfer during axial loading. As confirmed by the manufacturer, Ceramic Bond contains 10-MDP (<5 vol%) in addition to 3-MPTS; 10-MDP provides the chemically relevant phosphate functionality for an interaction with zirconia, whereas 3-MPTS acts as a silane for silica-containing ceramics. Ceramic Bond was therefore used as an MDP-containing multi-purpose primer.

GI was selected as the conventional comparator because conventional cementation of high-strength zirconia on retentive preparations remains clinically relevant, particularly in posterior regions where moisture control may be challenging and the straightforward removal of excess cement is advantageous. Clinical studies have reported acceptable outcomes for zirconia crowns cemented with GI under such conditions [21,22,23,24] and laboratory investigations have demonstrated adequate fracture loads [25,26]. Nevertheless, GI is not indicated for short or poorly retentive preparations, or in situations requiring maximal bond strength.

From a clinical perspective, these findings support a selective approach to cementation. For conventionally milled 3Y-TZP crowns on retentive preparations, adhesive cementation may provide an additional mechanical safety margin, particularly in high-load posterior regions. For current-generation 3D-printed zirconia, however, the present data do not demonstrate a mechanical advantage of adhesive cementation over GI under the tested conditions. Rather than establishing equivalence, this result suggests that any adhesive benefit may be smaller than the pronounced effect observed for milled zirconia and may be masked by the greater variability in the 3D-printed material.

### 4.2. Variability and Microstructural Considerations in Printed Zirconia

The broader IQRs and greater scatter observed in the 3D-printed groups, particularly after conventional cementation, indicate greater dispersion than in the milled controls. This interpretation is consistent with our previous investigations of the same LCM 3Y-TZP system, in which 3D-printed specimens showed substantially lower Weibull moduli (m ≈ 3–6) than milled specimens (m ≈ 14–16), despite comparable mean strengths. SEM analysis in those studies revealed interparticle and interlayer pores, binder-related voids, and flaw clusters near support interfaces that were absent or less pronounced in the milled controls [16,27,32].

Several process-related mechanisms may contribute to these defect populations. The high viscosity of zirconia slurry can impede the escape of entrapped air and thereby promote pore formation [29,35,36,37]. Layer interfaces may act as weak points, while non-uniform shrinkage during debinding and sintering may generate residual stresses. Incomplete binder removal, particulate contamination, and surface damage caused by support removal may introduce further microstructural irregularities [7,8,29,38,39,40,41]. The absence of a controlled clean-room environment in the present study may also have permitted contamination during printing or post-processing. Under load, pores and surface flaws can become preferential sites for crack initiation and propagation [29]. Independent X-ray-based investigations of LCM zirconia have reported pores predominantly in the 7–20 µm range, with maximum dimensions of approximately 54 µm and distributions influenced by the printing direction [29,35,40,41].

These limitations do not negate the potential advantages of additive manufacturing. Zirconia printing offers considerable geometric freedom and may facilitate thin restorations because no machining-related tooling stress is introduced. Further improvements in slurry conditioning, including controlled warming to reduce viscosity, cleaner manufacturing conditions, optimized support placement and removal, and refined debinding and sintering protocols, may reduce defect formation and improve reliability [42].

### 4.3. Manufacturing Reproducibility and Test-Related Factors

The printing orientation and support strategy were selected to preserve occlusal morphology, minimize staircase artifacts at functional contacts, and confine support-removal scars to nonfunctional axial surfaces. Column supports were therefore concentrated on the mesio-lingual aspect and kept away from cusp tips and fossae. Nevertheless, orientation and support placement can affect local cure depth, green-body stiffness, and shrinkage anisotropy. Moreover, the unpolished support scars, although positioned away from the loading contacts, remained potential surface flaws and may have contributed to variability if indirectly engaged by the load path.

All 3D-printed crowns were produced across two print builds and sintered in a single furnace cycle. This approach improved internal consistency but restricted the assessment of build-to-build and furnace-to-furnace variability, including differences in exposure uniformity, vat aging, slurry rheology, build-plate position, green-body handling, furnace loading, and thermal gradients. Consequently, the results cannot represent the full process variability expected across laboratories or manufacturing runs.

Methodological aspects of the test setup may also have affected the measured fracture loads. Airborne-particle abrasion of the 3D-printed resin dies may have caused heterogeneous material loss, altered the internal fit, and increased cement film thickness. An increased cement thickness may be particularly relevant for conventionally cemented restorations because these cements have lower compressive strength [43]. In addition, the 1 mm-thick tin foil used during load application enlarged the effective contact area and reduced local stress peaks. This approach improves reproducibility and limits direct damage by the indenter, but it may delay crack initiation, increase the recorded fracture load relative to direct-contact loading, and impede the precise localization of the fracture origin.

The use of standardized resin dies, identical specimen positioning, and a consistent loading procedure enhanced comparability between groups. However, the elastic modulus of the resin dies differs from that of natural dentin. Furthermore, although the specimens underwent thermocycling and mechanical loading before the terminal load-to-fracture test (6000 thermal cycles between 5 and 55 °C with a 2 min dwell time and 1.2 × 10^6^ load cycles at 50 N in distilled H_2_O), this pre-aging protocol does not replace staircase fatigue-to-failure testing. The final predominantly axial load also represents a simplified condition compared with the multidirectional and frequently oblique forces acting in vivo.

### 4.4. Limitations and Future Directions

Several limitations should therefore be considered. The modest sample size limited the detection of medium or small effects, particularly in the more variable 3D-printed groups. Only one zirconia formulation per manufacturing method, one adhesive primer/cement system, and one GI formulation were evaluated. Cementation was performed under controlled laboratory conditions and did not reproduce clinical contamination or operator-related variability. The high absolute fracture loads should, consequently, not be interpreted as direct clinical thresholds, but as relative measures for comparing the manufacturing and cementation protocols under standardized conditions.

No nondestructive porosity mapping by micro-CT or SEM fractography was performed on the crowns tested in the present study. Therefore, porosity, flaw size and location, grain-level features, and their relationships to the fracture origins could not be quantified for this specific cohort. Support-scar dimensions and surface roughness were likewise not measured. These omissions limit the extent to which the absence of a detectable adhesive benefit in 3D-printed crowns can be attributed to particular defect populations or processing variables.

Only a gross post-test crown failure classification was available; no systematic fractographic analysis was performed, and fracture origins and crack-propagation pathways could not be determined.

Future investigations should therefore (i) distribute specimens across multiple material lots, print builds, and furnace loads, and incorporate Batch/Build/Furnace as blocking or random factors in mixed-effects models; (ii) perform pre-test micro-CT followed by SEM/EDS and fractography to relate pore volume, defect size and location, and fracture origins to fracture load and Weibull parameters; (iii) compare alternative printing orientations and polished versus unpolished support scars; and (iv) evaluate additional adhesive systems and surface treatments, including different airborne-particle abrasion parameters and phosphate ester monomer concentrations. The influence of the supporting substrate should be examined using a stiffer dentin analog or natural dentin. Finally, staircase fatigue-to-failure testing after thermomechanical aging would provide a more clinically relevant assessment than terminal static loading alone. These measures will clarify whether improvements in printing and post-processing shift the dominant failure mechanism toward the crown–cement interface, where adhesive strategies may then yield gains comparable to those observed for milled zirconia.

## 5. Conclusions

Adhesive luting significantly increased fracture load for milled zirconia crowns. For 3D-printed zirconia, no statistically significant difference was detected between adhesive and conventional cementation under the present conditions. Given the variability and *n* = 8 per group, the study was powered to detect very large effects but not medium or small ones; therefore, the 3D-printed group result should not be interpreted as proof of no effect. The 3D-printed crowns showed broader IQRs and greater scatter, consistent with lower reliability. Future studies should incorporate multi-batch production, include Batch/Build/Furnace as blocking or random factors, and pair mechanical testing with nondestructive defect mapping and staircase fatigue-to-failure to quantify any cement-dependent effects in additively manufactured zirconia.

## Figures and Tables

**Table 1:** Mean fracture forces of 3D-printed and milled zirconia crowns: standard deviation (SD), 95% confidence interval (CI), interquartile range (IQR), and gross post-test crown failure patterns of 3D-printed and milled zirconia crowns.

		3D-Printed		Milled	
**Fracture load**		**Adhesive**	**Conventional**	**Adhesive**	**Conventional**
**Mean ± SD [N]**		4986 ± 1301	5581 ± 1618	7162 ± 1294	4021 ± 1126
**Min [N]**		2930	3126	4814	2289
**Max [N]**		6448	8207	8683	6275
**95% CI [N]**		3898; 6074	4228; 6933	6080; 8244	3080; 4963
**IQR [N]**		2195	2416	2015	881
**Gross post-test failure pattern** *	Crown fracture with fragment separation, *n*	8	8	7	8
	Crown crack without fragment separation, *n*	0	0	1	0

* Gross post-test failure patterns refer exclusively to the crowns. The extent of crown fragmentation and damage to the resin dies was not systematically assessed. No fracture origin or crack-propagation pathway was determined.

## Data Availability

The data presented in this study are available from the corresponding author upon reasonable request.
